# EphA5 protein, a potential marker for distinguishing histological grade and prognosis in ovarian serous carcinoma

**DOI:** 10.1186/s13048-016-0292-1

**Published:** 2016-11-25

**Authors:** Xiao Chen, Xuan Wang, Xue Wei, Jiandong Wang

**Affiliations:** Department of Pathology, Jinling Hospital, Nanjing University School of Medicine, Nanjing, 210002 China

**Keywords:** EphA5, Receptor tyrosine kinase, Ovarian serous carcinoma

## Abstract

**Background:**

Ovarian serous carcinoma (OSC) is the most common ovarian epithelial malignancy. Disregulation of Eph/ephrin signaling has been implicated in oncogenesis and tumor progression. EphA5 receptor is one of large families of Eph tyrosine kinase receptor and is documented in the development of nervous system. Till now, there is no published data about the role of EphA5 in ovarian epithelial neoplasmas.

**Methods:**

This study aims to investigate the expression of EphA5 protein in ovarian serous carcinoma, and its relationship to clinical pathological characteristics. Sixty-one cases of ovarian serous carcinoma, 24 cases of benign ovarian serous tumors, 42 cases of serous borderline tumors and 20 cases of normal fallopian tubes were examined using immunohistochemical staining. The relationship between EphA5 expression and pathological parameters was analyzed. Kaplan-Meier survival function was used to analyze prognosis of patients.

**Results:**

Immunostaining analysis demonstrated that the EphA5 protein was highly expressed in 100% (20/20) of normal fallopian tube samples, 100% (24/24) of benign epithelial ovarian tumors, 76% (32/42) of ovarian serous borderline tumors, and 31% (19/61) of ovarian serous carcinomas. Loss of EphA5expression was associated with tumor grade (*P* < 0.001) and FIGO stage (*P* = 0.005). The survival analysis showed that patients with negative or weak expression of EphA5 protein had a poor outcome than those with positive expression (*P* = 0.004).

**Conclusions:**

Our results show that EphA5 may be a potential biomarker for distinguishing high-and low-grade ovarian serous carcinoma and a potential prognostic marker.

## Background

Epithelial ovarian cancer (EOC) is the most lethal gynecological malignancy and the fifth common cause of tumor-related death in women in the United States. The survival rates for advanced stage of the disease that have not changed in several decades [1, 2]. Lack of specific symptoms of the disease in its early stages is a significant factor contributing to the high mortality rate. Consequently, most patients have reached advanced stage at the time of diagnosis [3]. Despite new diagnostic and surgical advances, and development of chemotherapeutic regimens, the overall 5-year survival for women with advanced stage epithelial ovarian cancer has remained approximately 12% over the past 40 years [4]. Ovarian serous carcinoma (OSC) is a common ovarian epithelial malignancy, which has traditionally been graded as high, moderately, and poorly differentiated, indicating that it is a homogeneous disease in the position of pathogenesis. More recently, a 2-tier grading system has been proposed, in which ovarian serous carcinomas are subdivided into low-grade and high-grade. Seminal clinicopathologic and molecular genetic studies have revealed that this approach is simplistic, reproducible, and on the basis of underlying morphologic and molecular genetic differences between low-grade and high-grade tumors [5]. The progression to invasive carcinoma is a slow step-wise process. Low-grade tumors are indolent and have better outcome than high-grade tumors [6]. Notwithstanding the enormous effort, the etiopathogenesis of epithelial ovarian cancers are still unknown. The development of more effective therapeutic strategies highlight the need to better understand the molecular mechanisms of ovarian cancer.

The Eph (Erythropoietin-producing human hepatocellular carcinoma cell) family of receptors and ligands is the largest group of tyrosine kinase receptor-ligand systems, which is involved in many physiological roles including axon guidance, neural plasticity, angiogenesis, cell migration and tissue segmentation [7, 8]. Based on the structural homology and the binding affinities, The Eph receptors and their ephrin ligands are classified into two groups, A and B. Ephrin-A ligands generally bind to EphA receptors via a glycosylphosphatidylinositol anchor on the cell membrane, whereas ephrin-B ligands bind to EphB receptors via a transmembrane domain [9]. Eph receptors have been implicated in mediating developmental events, particularly in the nervous system. The roles of the Eph receptors and ephrin ligands in cell adhesion, migration, formation of the borders of compartments, regulation of cell proliferation in various tumors and angiogenesis are also well documented [10, 11]. EphA5 (also known as REK7, Ehk-1, Bsk) is a member of the Eph receptor tyrosine kinase subfamily, originally identified as a nervous-system-specific orphan receptor expressed in embryonic rats at high levels and in the adult brain at lower levels [12]. The function of the EphA5 receptor is well established as an axon guidance molecule during neural system development [13]. However, the potential role of EphA5 in human carcinogenesis has not been well addressed. Fu et al. revealed that increased methylation of EphA5 is correlated with decreased expression in primary breast cancer [14]. Giaginis et al. found that pancreatic adenocarcinoma cases with enhanced EphA5 expression presented significantly increased tumor cells proliferative capacity [15]. To date, there have been no published reports describing the role of EphA5 expression in epithelial ovarian carcinoma. Here, we evaluated the expression of EphA5 protein in a set of normal fallopian tube, benign epithelial ovarian tumors, ovarian serous borderline tumors, and ovarian serous carcinomas samples to explore its roles in ovarian serous carcinoma.

## Methods

### Patients and clinicopathological variables

The study cohort consisted of 61 patients with ovarian serous carcinoma (age range 25–69 years, mean age 50 years; high-grade 40, low-grade 21), 24 patients with benign ovarian serous tumors (age range 23–62 years, mean age 34 years), 42 patients with ovarian serous borderline tumors (age range 19–45years, mean age 33 years) and 20 normal fallopian tube tissue samples obtained from patients (age range 46–57 years, mean age 47 years) who underwent surgery for benign gynecological diseases from 2005 to 2014 in Jinling Hospital, Nanjing, China. All hematoxylin and eosin stained slides were reviewed by two pathologists to verify the diagnosis, histological grade and stage. Pathological stage and histological subtype were determined for each surgical specimen according to the 2002 International Federation of Gynecology and Obstetrics (FIGO) criteria, and Pathology and Genetics Tumors of the Breast and Female Genital Organs (World Health Organization, WHO 2003). A two-tier (low-grade and high-grade) system was used to define the differentiation of ovarian serous carcinomas. Patient data were obtained from hospital tumor registry and telephone review. None of the patients received preoperative chemotherapy or radiation therapy. All cases of recurrence had radio-graphic evidence of disease or biopsy proven progression of disease. Sixty-one patients with ovarian serous carcinoma were followed up until April 2015. The records of patients who were alive at follow-up or who did not die of disease were considered to be censored. Ethical approval was obtained from Ethics Committee of Jinling Hospital, Nanjing, China (2016NZGKJ-075).

### Immunohistochemistry

**Fig. 1:**
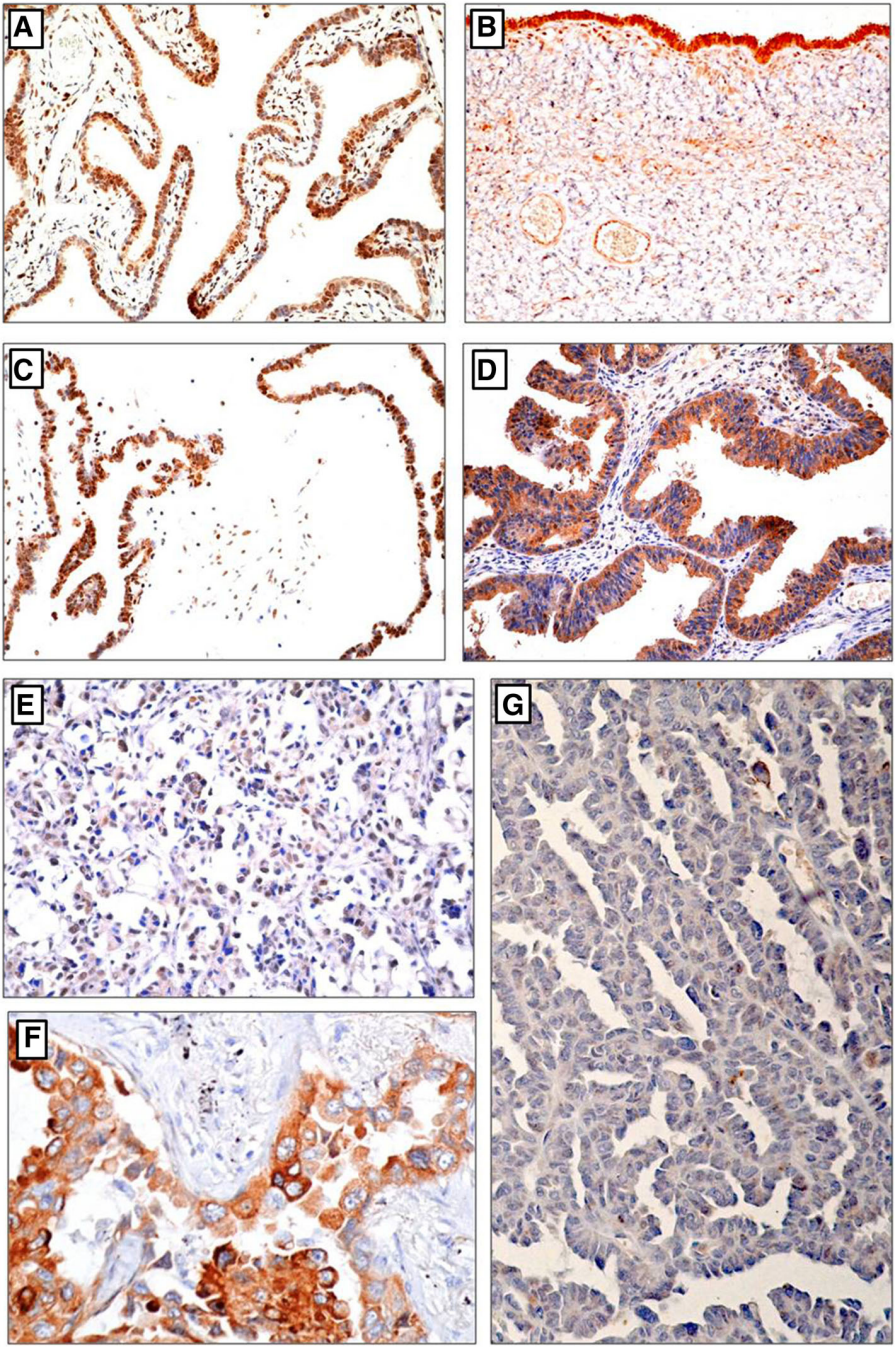
Expression of EphA5 in normal fallopian tube and ovarian serous tumors. **a** High expression of EphA5 in normal fallopian tube. **b** High expression of EphA5 in benign serous tumor. **c** High expression of EphA5 in serous borderline tumor. **d** High expression of EphA5 in low-grade serous carcinoma. **e** Low expression of EphA5 in high-grade serous carcinoma. **f** Positive control (lung adenocarcinoma). **g** Negative control

**Fig. 2:**
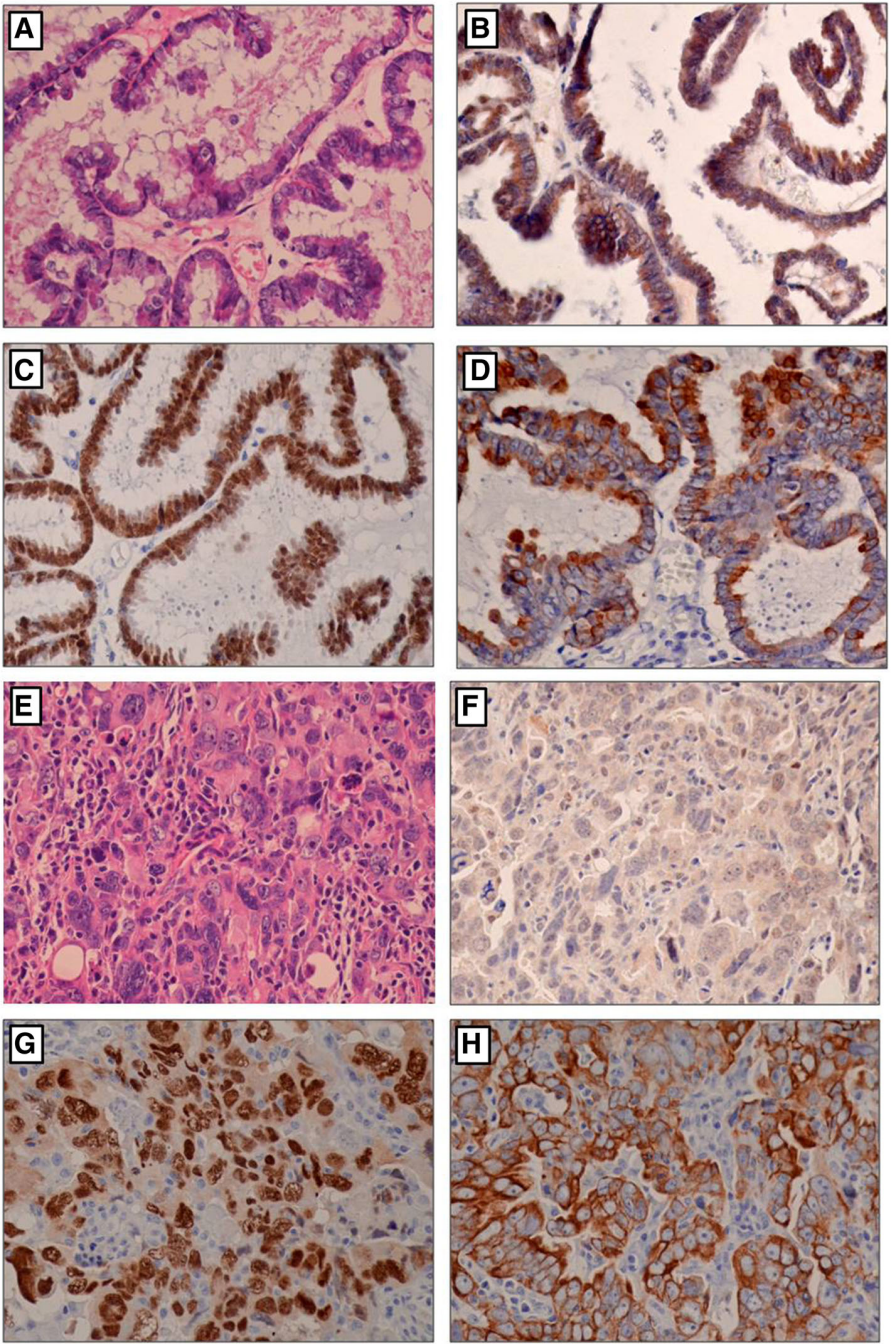
Representative examples of expression of EphA5, PAX8 and cytokeratin in low- and high-grade ovarian serous carcinomas. **a** and **e**: **h** and **e** staining of low- and high-grade ovarian serous carcinoma. **b** and **f**: Staining of EphA5; **c** and **g**: staining of PAX8; **d** and **h**: staining of cytokeratin in low- and high-grade ovarian serous carcinoma

Sections from surgical specimens were fixed in 10% formalin and embedded in paraffin and were used for immunohistochemical staining (IHC) according to a standard method. Briefly, each 4-μm tissue section was deparaffinized and rehydrated. After rehydration through a graded ethanol series, the sections were autoclaved in 10 mM citrate buffer (pH 6.0) at 120 °C for 2 min for antigen retrieval, then cooled to 30 °C and washed with phosphate-buffered saline (PBS, pH 7.3). After endogenous peroxidase had been quenched with aqueous 3% H_2_O_2_ for 10 min, the sections were washed with PBS, incubated at 4 °C overnight with primary rabbit polyclonal anti-EphA5 antibody (Abgent, San Diego, CA, USA) at a dilution of 1:600 and then washed with PBS. The sections were incubated with secondary antibody (Dako REAL EnVision Detection System, Dako, UK) for 30 min at room temperature. This was followed by color development with 3, 3′-diaminobenzidine (DAB) solution for 1 min. Nuclei were lightly counterstained with hematoxylin. The lung cancer with known positivity was used as a positive external control (Fig. 1f). Primary antibody was replaced with antibody diluent for negative controls (Fig. 1g). PAX8 and cytokeratin protein were checked in high and low grade serous ovarian carcinomas (Fig. 2).

EphA5 staining was independently evaluated for immunoreactivity by two pathologists who were double-blinded to clinical data according to the scoring criteria. Immunohistochemical staining of cancer cells was assessed according to the staining intensity of positive cells. EphA5 expression was assessed for intensity (0 = no staining, 1 + = weak, 2 + = moderate, 3 + = strong).

### Statistical analysis

The statistical significance of intergroup differences was evaluated by Spearman correlation analysis. Survival curves were constructed using the Kaplan-Meier method and the differences between the curves were compared by the log-rank test. A multivariate Cox proportional hazards model was used to analyze independent influence of EphA5 gene in survival. *P*-values <0.05 (two-sided) were considered statistically significant. All analyses were performed by SPSS software (version 16.0, Chicago, IL).

## Results

### EphA5 expression in fallopian tube, ovarian benign, borderline and serous carcinoma

The expression of EphA5 protein was determined in ovarian epithelial tumors and normal fallopian tube by immunohistochemical staining (Fig. 1). As shown in Figs. 1 and 2, EphA5 staining was predominantly localized in the cytoplasm. High and moderate expression of EphA5 was observed in 100% (20/20) of normal fallopian tube samples, 100% (24/24) of benign epithelial ovarian tumors, 76% (32/42) of ovarian serous borderline tumors, and 31% (19/61) of ovarian serous carcinomas. The expression of EphA5 protein was significantly decreased from normal fallopian tubes, benign ovarian serous tumors, ovarian serous borderline tumors to ovarian serous carcinomas (*P* < 0.001) (Table 1).

**Table 1:** The expression of EphA5 in fallopian tube, ovarian benign tumor, border line tumor and serous carcinoma

Parameters	No.	EphA5 protein expression			*P* value
	147	0/1+	2+	3+	
Fallopian tube	20	0	0	20	
Benign tumor	24	0	1	23	
Borderline tumor	42	10	25	7	
Ovarian serous carcinoma	61	42	15	4	<0.001

### EphA5 expression in ovarian serous carcinoma and its correlation with clinicopathological parameters

EphA5 protein was differentially expressed in 61 samples of ovarian serous carcinoma. Forty-two of 61 (68.9%) samples were negatively or weakly (0/1+) stained with anti-Eph A5 antibody, 15 of 61samples (24.6%) were moderately stained (2+) and 4 of 61 samples (6.6%) were strongly stained (3+). The expression of EphA5 was decreased in ovarian serous carcinoma compared with normal fallopian tube. The relationship between EphA5 expression and pathological parameters was analyzed (Table 2). Expression of EphA5 was significantly associated with grade (*P* < 0.001) and FIGO stage (*P* = 0.005). No significant relationship was observed between the expression of EphA5 and age (*P* = 0.178), diameter (*P* = 0.140) and metastasis (*P* = 0.168).

**Table 2:** The relationship between the expression of EphA5 and clinicopathological parameters of ovarian serous carcinoma

Parameters	No.	EphA5 protein expression			*P* Value
	61	0/1+	2+	3+	
FIGO Stage					0.005
I + II	26	13	10	3	
III + IV	35	29	5	1	
Grade					
high	40	38	2	0	<0.001
low	21	4	13	4	
Diemeter (cm)					0.140
≤ 5	16	13	3	0	
5–10	32	21	7	4	
> 10	13	7	6	0	
Age (years)					0.178
< 50	29	17	11	1	
≥ 50	32	25	4	3	
Metastasis					0.168
Yes	37	28	8	1	
No	17	7	8	2	
Not available	7	6	0	1	

### Association between EphA5 expression and overall survival in patients with serous ovarian cancer

**Fig. 3:**
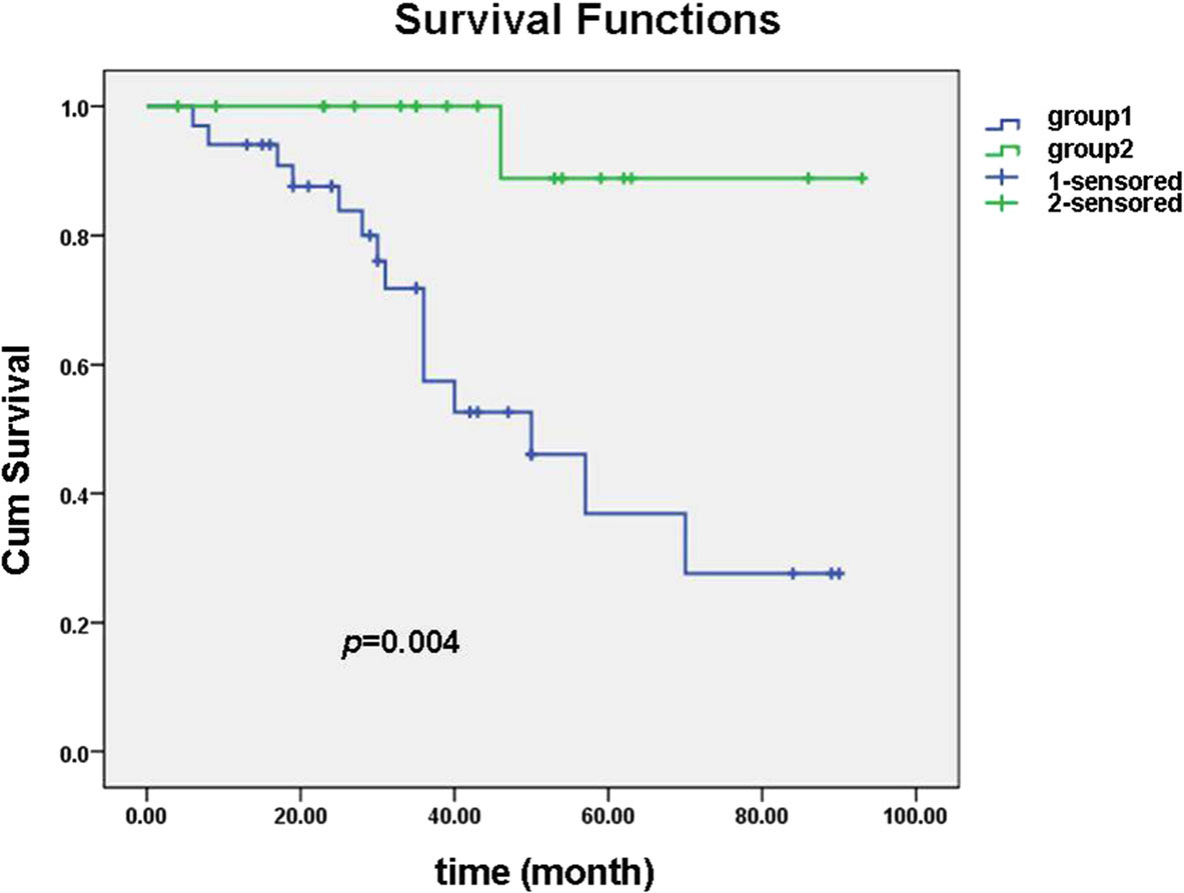
Kaplan-Meier plots of overall survival show patients with loss of the expression of EphA5 had a shorter survival than those with high expression of EphA5. (group 1: 0 or 1+ of EphA5; group 2: 2+ and 3+ of EphA5)

Fifty-three cases stained for EphA5 had follow-up data for survival and were available for assessment. Using the follow-up data of the 53 patients in conjunction with the results from the EphA5 IHC staining experiments, we showed that patients with high expression had a significantly favorable overall survival (OS) compared to patients with negative or weak expression (*P* = 0.004, Fig. 3). Data from Cox proportional hazards model indicated that EphA5 expression (*P* = 0.049, *HR* = 2.532, 95% *CI* = 0.104–2.722) and FIGO stage (*P* = 0.006, *HR* = 5.290, 95% *CI* = 1.413–7.659) are independent influences in prediction of survival.

## Discussion

EphA5 plays a role in spine formation and synaptogenesis. In contrast to these more established roles, EphA5 function in cancer is much less clear. Fu et al. [14] found that the level of EphA5 mRNA was dramatically decreased in 5 breast cancer cell lines. Previously, we detected the EphB6 protein in ovarian serous carcinoma. Decreased expression of EphB6 was found in ovarian serous carcinomas compared with that in normal fallopian tubes. The loss of EphB6 protein was associated with higher tumor grade and TNM stage [16]. In current study, we found that loss of expression of EphA5 was more often found in high-grade and advanced FIGO stage in ovarian serous carcinoma. Women with low-grade serous ovarian carcinoma are younger and have longer survival than women with high-grade tumors. It is crucial to separate women with serous ovarian carcinoma into 2 clinically distinct populations, low-grade and high-grade, as they deserve unique consideration of and treatment for their specific histotypes [17–19]. Our findings that EphA5 was absent in high-grade serous ovarian carcinoma indicate that EphA5 may be a new biomarker for distinguishing high- and low-grade ovarian serous carcinoma. The data was consistent with the survival analysis that negative or weak expression of EphA5 protein had a poorer outcome than those with high expression.

DNA methylation at promoter region is a powerful epigenetic modification regulates gene transcription. EphA5 gene silenced by methylation has been demonstrated in breast cancer [14], prostate cancer [20], and colorectal cancer [21], implying that the hypermethylation of EphA5 might be of great importance during tumorigenesis and progression in cancer. Identification of hypermethylated EphA5 DNA in ovarian serous carcinoma will improve our understanding of the mechanisms leading to the downregulation of EphA5 expression in ovarian tumorigenesis and can serve as valuable diagnostic marker. In our next study, we will determine the relationship between expression level and the methylation status of EphA5 in ovarian cancer tissues.

It is now widely accepted that low-grade and high-grade serous tumors are essentially distinct diseases. They exhibit distinct genetic alterations, molecular patterns and clinical behavior. Clinically, high-grade serous carcinoma are aggressive neoplasms which frequently affect women in the perimenopausal or postmenopausal age group, while low-grade serous carcinomas are relatively indolent and affect younger women. Low-grade serous carcinomas are more refractory to platinum-based chemotherapy than high-grade serous carcinoma. High-grade and low-grade serous carcinomas are usually easily distinguished, it may be difficult when nuclear features are intermediate between grade 1 and grade 3; and in small tissue samples.

## Conclusions

Our results show that EphA5 is a potential biomarker for distinguishing high-and low-grade ovarian serous carcinoma, and a potential prognostic marker in ovarian serous carcinomas.

## Abbreviations

EOC: Epithelial ovarian cancer
Eph: Erythropoietin-producing human hepatocellular carcinoma cell
IHC: Immunohistochemical staining
OSC: Ovarian serous carcinoma
